# Health Care Resource Utilization for Patients With Suspected Myocardial Infarction: A Secondary Analysis of the RACE-IT Randomized Clinical Trial

**DOI:** 10.1001/jamanetworkopen.2025.6930

**Published:** 2025-04-25

**Authors:** Joseph Miller, Bernard Cook, Satheesh Gunaga, Raef Fadel, Chaun Gandolfo, Joshua Emakhu, Nicholas L. Mills, Simon Mahler, Phillip Levy, Sachin Parikh, Seth Krupp, Kegham Hawatian, Khaled Nour, Howard Klausner, Ryan Gindi, Michael Hudson, Giuseppe Perrotta, Bryan Zweig, David Lanfear, Henry Kim, Shooshan Danagoulian, Catriona Keerie, Hashem Nassereddine, Thayer Morton, Ziad Affas, Arqam Husain, James McCord

**Affiliations:** 1 Department of Emergency Medicine, Henry Ford Health + Michigan State University Health Sciences, Detroit; 2 Department of Pathology, Henry Ford Health + Michigan State University Health Sciences, Detroit; 3 Heart and Vascular Institute, Henry Ford Health + Michigan State University Health Sciences, Detroit; 4 British Heart Foundation Center for Cardiovascular Science, The University of Edinburgh, Edinburgh, United Kingdom; 5 Department of Emergency Medicine, Wake Forest University School of Medicine, Winston-Salem, North Carolina; 6 Department of Emergency Medicine, Wayne State University, Detroit, Michigan; 7 College of Liberal Arts and Sciences, Wayne State University, Detroit, Michigan; 8 College of Medicine and Veterinary Medicine, The University of Edinburgh, Edinburgh, United Kingdom; 9 Department of Emergency Medicine, Corewell Health Beaumont Hospital, Royal Oak, Michigan; 10 Department of Cardiology, Ascension Providence, Southfield, Michigan

## Abstract

**Importance:**

Evaluation for myocardial infarction (MI) in emergency departments (EDs) is a common, resource-intensive process. High-sensitivity cardiac troponin I (hs-cTnI) assays have become a key tool in rapidly ruling out MI, with the potential to reduce health care resource utilization.

**Objective:**

To determine whether a 0-hour and 1-hour (hereafter referred to as 0/1-hour) hs-cTnI accelerated protocol reduces health care resource utilization compared with a traditional 0/3-hour standard care protocol for MI exclusion in the ED.

**Design, Setting, and Participants:**

This is a prespecified secondary analysis of the RACE-IT trial, a stepped-wedge randomized clinical implementation trial conducted across 9 EDs in Michigan. The trial enrolled 32 608 consecutive ED patients evaluated for suspected MI between July 8, 2020, and April 3, 2021. Statistical analysis was conducted from July 10 to September 5, 2024.

**Interventions:**

The 0/1-hour hs-cTnI accelerated protocol for MI exclusion was compared with the traditional 0/3-hour standard care protocol.

**Main Outcomes and Measures:**

Main outcomes were ED discharge to home, ED length of stay, rates of cardiac stress testing, cardiology consultation, left heart catheterization, and cardiac revascularization within 30 days.

**Results:**

A total of 32 608 patients (median age, 59 years [IQR, 45-71 years]; 18 705 women [57.4%]) were included in the analysis. The rate of ED discharge to home was 58.0% for the accelerated protocol group (11 082 of 19 103) and 59.8% for the standard care group (8070 of 13 505) (adjusted odds ratio [AOR], 1.05; 95% CI, 0.95-1.15). The accelerated protocol group showed significant reductions in the odds of cardiac stress testing (3.3% [623 of 19 103] vs 3.9% [526 of 13 505]; AOR, 0.62; 95% CI, 0.49-0.78), cardiology consultations (8.6% [1640 of 19 103] vs 12.2% [1651 of 13 505]; AOR, 0.57; 95% CI, 0.49-0.67), and left heart catheterization rates (1.0% [198 of 19 103] vs 1.2% [167 of 13 505]; AOR, 0.65; 95% CI, 0.43-0.99) compared with the standard protocol group. The median ED length of stay decreased by 20 minutes (IQR, 18-24 minutes) in the accelerated protocol group, with no significant change in revascularization rates.

**Conclusions and Relevance:**

This secondary analysis of a randomized clinical trial of a 0/1-hour hs-cTnI protocol to rule out MI in the ED found that there was a reduction in cardiac evaluations and ED length of stay without increasing revascularization rates compared with the standard 0/3-hour hs-cTnI protocol. This approach could optimize health care resources in EDs.

**Trial Registration:**

ClinicalTrials.gov Identifier: NCT04488913

## Key Points

**Question:**

Does implementing a 0-hour and 1-hour (hereafter referred to as 0/1-hour) high-sensitivity cardiac troponin I (hs-cTnI) protocol reduce health care resource utilization compared with a 0/3-hour standard protocol for myocardial infarction exclusion in US emergency departments (EDs)?

**Findings:**

In a secondary analysis of this stepped-wedge randomized clinical trial including 32 608 patients with suspected myocardial infarction, a 0/1-hour hs-cTnI protocol reduced cardiac testing and the median length of ED stay without changing rates of ED discharge or revascularization.

**Meaning:**

This study suggests that a 0/1-hour hs-cTnI protocol can reduce health care resource utilization and ED crowding without compromising safety.

## Teaser

This secondary analysis of a randomized clinical trial assesses whether a 0-hour and 1-hour high-sensitivity cardiac troponin I protocol reduces health care resource utilization compared with a traditional 0-hour and 3-hour protocol for myocardial infarction exclusion in the emergency department.

## Introduction

Evaluation for possible myocardial infarction (MI) is one of the most common diagnostic assessments performed in US emergency departments (EDs).^1^ Strategies to evaluate patients for possible MI are historically costly, are associated with significant resource utilization, and can contribute to ED overcrowding.^2,3,4^ Cardiac troponin (cTn) is the cornerstone for detecting MI, and cTn assays have greatly improved in sensitivity and precision.

Current American College of Cardiology/American Heart Association guidelines recommend the use of high-sensitivity cTn (hs-cTn) to rapidly rule out MI.^5^ High-sensitivity cTn assays, whether using hs-cTnT or high-sensitivity cardiac troponin I (hs-cTnI), allow for precise quantification of cTn and have been successfully incorporated into accelerated protocols to exclude MI.^6,7,8^ Trials evaluating accelerated protocols have safety comparative with that of traditional strategies,^9,10,11^ and guidelines now recommend implementing accelerated protocols over 1 to 2 hours with consideration of discharging patients at low risk from the ED without further testing.^5,12^

Although discharging patients at low risk using accelerated protocols has the potential to reduce health care resource utilization, there is concern that reporting low hs-cTn values below the 99th percentile could increase placements in observation units (short stay units), cardiac testing, and revascularization procedures.^13,14^ Furthermore, most studies of accelerated protocols have been performed outside of the US or within the context of highly selective clinical trials. Using a large implementation trial within a US health system,^15^ we planned a secondary analysis to assess the effect of an hs-cTnI accelerated protocol on health care resource utilization compared with a traditional approach.

## Methods

### Study Design and Population

RACE-IT (Rapid Acute Coronary Syndrome Exclusion Using High-Sensitivity I Cardiac Troponin) was a stepped-wedge randomized clinical trial enrolling consecutive patients evaluated for possible MI in 9 EDs within Henry Ford Health in Michigan. The trial protocol and primary results are published^15,16^ (trial protocol and statistical analysis plan in Supplement 1). Eligibility was broad and included clinical suspicion for MI, as evidenced by the clinician ordering any troponin and electrocardiogram testing. Exclusion criteria were age younger than 18 years, ST-segment elevation MI, troponin levels higher than the 99th percentile during the first 3 hours, trauma, transfers from another facility, residence outside Michigan, and hospice enrollment. We excluded patients with troponin levels higher than 99th percentile, as their evaluation and management would not differ from a traditional approach. Patients were enrolled only once, and the study enrollment period was from July 8, 2020, to April 3, 2021. Participants’ race and ethnicity were included in the study as self-reported and classified according to categories within the electronic health record system (EPIC), including African American or Black, Asian, Hispanic or Latinx, White, and other (Hawaiian Pacific Islander and Native American) or unknown. Race and ethnicity were assessed in this study due to known differences among races and ethnicities in cardiovascular disease. The Henry Ford Health institutional review board approved the trial and granted a waiver of consent because data were deidentified. This study followed the Consolidated Standards of Reporting Trials (CONSORT) reporting guideline.

### Randomization and Intervention

The order in which each of the EDs implemented the accelerated protocol was randomly assigned. Eligible patients were included for study analysis at all times during the trial period except a 3-week transition phase when the accelerated protocol was first introduced at a site to enable familiarization with the protocol (eFigure 1 in Supplement 2).

In the standard care cohort, MI was excluded if all cTn values were below the 99th percentile at 0 and 3 hours. Clinicians could not view concentrations below the 99th percentile. Clinicians considered patients appropriate for early discharge without further cardiac testing if they had MI excluded at 3 hours and had a modified HEART (History, Electrocardiogram, Age, Risk factors, and Troponin) score (HEART [History, Electrocardiogram, Age, and Risk factors] score) less than 4.^17^

The accelerated protocol allowed for the exclusion of MI at 0 hours if the hs-cTnI value was less than 4 ng/L (to convert to micrograms per liter, multiply by 0.001) or at 1 hour if the 0-hour hs-cTnI value was 4 ng/L and the 1-hour value was less than 8 ng/L. All sites used a hs-cTnI assay (Access; Beckman Coulter), which has a 99th percentile value of 18 ng/L and a limit of quantitation of 0.8 ng/L.^18^ Sex-specific thresholds were not used. Patients with MI excluded at 0-hour and 1-hour (hereafter referred to as 0/1 hour) could be considered for early discharge from the ED without further cardiac testing, irrespective of their modified HEART score. Patients who were not ruled out within 1 hour had an additional hs-cTnI measurement at 3 hours, and they could be considered for discharge if their HEART score was less than 4 and all hs-cTnI values were 18 ng/L or less (eFigure 2 in Supplement 2). If a patient presented early (<3 hours after experiencing symptoms), the protocol instructed clinicians to use their best clinical judgement application of the accelerated protocol. Data on the time of symptom onset were not collected.

### Study Outcomes

Outcomes included ED discharge and ED length of stay. Outcomes also included cardiology consultation, cardiac stress testing, coronary computed tomography, left heart catheterization, and coronary revascularizations within 30 days of the initial ED encounter. Stress testing included exercise stress testing without imaging, stress testing with nuclear imaging (exercise or pharmacologic), or stress testing with echocardiography imaging (exercise or dobutamine). Revascularization included coronary artery bypass surgery or percutaneous coronary intervention. We analyzed the length of stay for patients discharged from the ED by different quantiles using quantile regression models. We supplemented information from the health system electronic health record (EPIC) with additional statewide data obtained through a health information exchange.^19^

### Statistical Analysis

Statistical analysis was conducted from July 10 to September 5, 2024. For this prespecified secondary analysis, we analyzed the entire cohort. Furthermore, we conducted studies for the subset of patients with a chief symptom of chest pain and for those who were ruled out early within 0/1 hour. These latter subgroup analyses were ad hoc and not planned in the primary trial protocol.

To compare the 2 arms, we used generalized linear mixed models, which were robust, to account for fixed and random effects and analysis of the categorical outcomes in this study. We used quantile regression models to analyze ED length of stay, assessing the 25%, 50%, and 75% quantiles. These models (unless otherwise specified) included the standard care vs accelerated protocol arm indicator as well as the following covariates: sex, age, racial and ethnic category (defined as Black, White, and other [Asian, Hawaiian Pacific Islander, Hispanic or Latinx, and Native American] or unknown), time since the initial study start date, coronary artery disease, and ED site (treated as a random effect). Quantile regression analysis included ED site as a random effect as well. All *P* values were from 2-sided tests and results were deemed statistically significant at *P* < .05. We performed all analyses using SAS, version 9.4 (SAS Institute Inc).

## Results

### Study Population Characteristics

The trial enrolled 32 608 patients (median age, 59 years [IQR, 45-71 years]; 18 705 women [57.4%] and 13 898 men [42.6]; and 9392 Black patients [28.8%], 19 415 White patients [59.5%], and 3801 patients of other or unknown race or ethnicity [11.7%]) (Table 1). There were 47 831 patients assessed for study eligibility, from which these 32 608 patients were eligible and enrolled. Of those eligible, 13 505 were in the standard care cohort, and 19 103 were in the accelerated protocol (Figure).

**Table 1.** Demographic and Clinical Characteristics of Study Population

Characteristic	All (N = 32 608)	Standard care (n = 13 505)	Accelerated protocol (n = 19 103)
Age, median (IQR), y	59 (45-71)	60 (46-73)	58 (45-71)
Sex, No. (%)			
Male	13 898 (42.6)	5701 (42.2)	8197 (42.9)
Female	18 705 (57.4)	7803 (57.8)	10 902 (57.1)
Unknown	5 (0.02)	1 (0.01)	4 (0.02)
Race and ethnicity, No. (%)			
Black	9392 (28.8)	3047 (22.6)	6345 (33.2)
White	19 415 (59.5)	9157 (67.8)	10 258 (53.7)
Other or unknown^a^	3801 (11.7)	1301 (9.6)	2500 (13.1)
Comorbid conditions, No. (%)			
Hypertension	15 602 (47.8)	6733 (49.9)	8869 (46.4)
Diabetes	7097 (21.8)	2959 (21.9)	4138 (21.7)
Hyperlipidemia	5619 (17.2)	2595 (19.2)	3024 (15.8)
Coronary artery disease	3627 (11.1)	1575 (11.7)	2052 (10.7)
Peripheral vascular disease	1329 (4.1)	546 (4.0)	783 (4.1)
Congestive heart failure	3502 (10.7)	1572 (11.6)	1930 (10.1)
Abdominal aortic aneurysm	269 (0.8)	139 (1.0)	130 (0.7)
Atrial fibrillation	2892 (8.9)	1345 (10.0)	1547 (8.1)
Chronic kidney disease	6647 (20.4)	2989 (22.1)	3658 (19.1)
Chronic lung disease	6692 (20.5)	2930 (21.7)	3762 (19.7)
Chest pain	9690 (29.7)	3952 (29.3)	5738 (30.0)
SARS-CoV-2 infection, No. (%)	1883 (5.8)	467 (3.5)	1416 (7.4)
Placed in observation, No. (%)	4308 (13.2)	1906 (14.1)	2401 (12.6)

^a^ Other race is inclusive of Asian, Hawaiian Pacific Islander, Hispanic or Latinx, and Native American.

**Figure.**
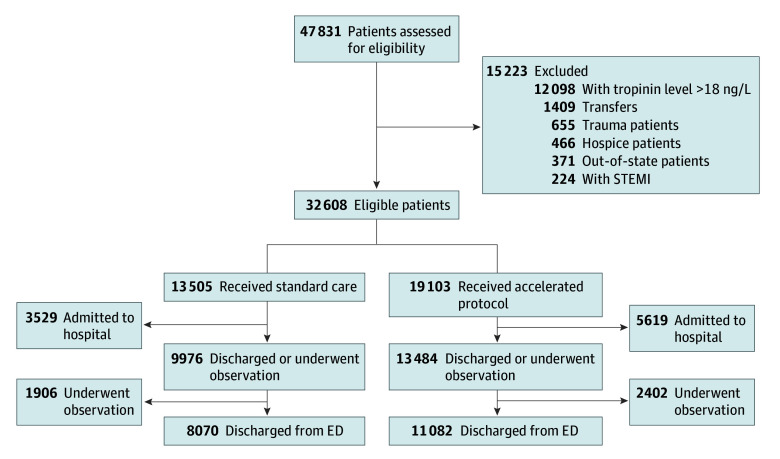
Flow Diagram of Protocol Allocation and Disposition From the Emergency Department (ED) STEMI indicates ST-elevation myocardial infarction. SI conversion factor: To convert troponin to micrograms per liter, multiply by 0.001.

Table 1 summarizes a detailed breakdown of study population demographic and clinical characteristics in both standard care and accelerated protocol cohorts. There was complete data collection on health care resources for the study cohort related to the patients’ encounters. Cardiac stress testing (1149 [3.5%]) and left heart catheterization (365 [1.1%]) procedures occurred infrequently in the overall cohort.

### Comparison of Resource Utilization for Total Study Population

Table 2 summarizes the differences in health care resource utilization between the accelerated protocol and standard care cohorts for the entire study population. In brief, there was no significant difference between the accelerated protocol and standard care cohorts in rates of ED discharge to home (58.0% [11 082 of 19 103] vs 59.8% [8070 of 13 505]; adjusted odds ratio [AOR], 1.05; 95% CI, 0.95-1.15). Unadjusted comparisons can be found in eTable 1 in Supplement 2. The accelerated protocol group showed significant reductions in the odds of cardiac stress testing (3.3% [623 of 19 103] vs 3.9% [526 of 13 505]; AOR, 0.62; 95% CI, 0.49-0.78), cardiology consultation (8.6% [1640 of 19 103] vs 12.2% [1651 of 13 505]; AOR, 0.57; 95% CI, 0.49-0.67), and left heart catheterization rates (1.0% [198 of 19 103] vs 1.2% [167 of 13 505]; AOR, 0.65; 95% CI, 0.43-0.99) compared with the standard protocol group. There was no significant difference in rates of revascularization.

**Table 2.** Overall Comparison of Health Care Resource Utilization

Outcome	Patients, No. (%)			AOR (95% CI)^a^	*P* value
	Overall (N = 32 608)	Standard care (n = 13 505)	Accelerated protocol (n = 19 103)		
ED disposition					
Discharged	19 152 (58.7)	8070 (59.8)	11 082 (58.0)	1.05 (0.95-1.15)	.35
Inpatient	9148 (28.1)	3529 (26.1)	5619 (29.4)		
Observation	4308 (13.2)	1906 (14.1)	2402 (12.6)		
Cardiac stress testing (any)	1149 (3.5)	526 (3.9)	623 (3.3)	0.62 (0.49-0.78)	<.001
Exercise ECG	30 (0.1)	17 (0.1)	13 (0.1)	0.97 (0.25-3.76)	.97
Stress echocardiography	288 (0.9)	149 (1.1)	139 (0.7)	0.53 (0.34-0.83)	.005
Nuclear imaging	846 (2.6)	369 (2.7)	477 (2.5)	0.64 (0.49-0.85)	.001
Cardiology consultation	3291 (10.1)	1651 (12.2)	1640 (8.6)	0.57 (0.49-0.67)	<.001
Coronary computed tomography	15 (0.1)	9 (0.1)	6 (0.03)	0.88 (0.15-5.14)	.89
Left heart catheterization	365 (1.1)	167 (1.2)	198 (1.0)	0.65 (0.43-0.99)	.04
Revascularization^b^	113 (0.4)	50 (0.4)	63 (0.3)	0.66 (0.32-1.36)	.26

Abbreviations: AOR, adjusted odds ratio; ECG, electrocardiography; ED, emergency department.

^a^ Covariates included sex, age, race and ethnicity, time since the initial study start date, history of coronary artery disease, and ED site (treated as a random effect).

^b^ Inclusive of percutaneous coronary intervention or coronary artery bypass surgery.

When comparing the subgroup of 9014 patients in the accelerated protocol cohort who were ruled out at 0 hours with the overall standard care cohort, these accelerated protocol patients had more significant reductions in health care resource utilization. Discharge rates were 76.9% (6932 of 9014) for the accelerated protocol group vs 59.8% (8076 of 13 505) for the standard care group (AOR, 1.75; 95% CI, 1.54-1.99). Stress testing rates were 2.8% (252 of 9014) for the accelerated protocol group vs 3.9% (526 of 13 505) for the standard care group (AOR, 0.66; 95% CI, 0.48-0.91), and cardiology consultation rates were 5.0% (451 of 9014) for the accelerated protocol group vs 12.2% (1651 of 13 505) for the standard care group (AOR, 0.53; 95% CI, 0.42-0.66).

### Comparison of Resource Utilization for Patients With Primary Symptom of Chest Pain

In an analysis restricted to patients with a primary symptom of chest pain, the adjusted odds of discharge from the ED under the accelerated protocol were higher than those under the standard care (AOR, 1.32; 95% CI, 1.07-1.63) (Table 3). The adjusted odds of cardiac testing and cardiology consultation were also reduced, similar to those seen for the entire study population.

**Table 3.** Comparison of Health Care Resource Utilization Among Patients With a Primary Symptom of Chest Pain

Outcome	Patients, No. (%) (N = 9690)		AOR (95% CI)	*P* value
	Standard care (n = 3952)	Accelerated protocol (n = 5738)		
Discharged from ED	2972 (75.2)	4373 (76.2)	1.32 (1.07-1.63)	.008
Cardiac stress testing (any)	282 (7.1)	420 (7.3)	0.74 (0.53-1.02)	.07
Exercise ECG	15 (0.4)	10 (0.2)	1.37 (0.31-6.02)	.68
Stress echocardiography	149 (3.8)	226 (3.9)	0.64 (0.41-1.00)	.047
Nuclear imaging	237 (6.0)	346 (6.0)	0.66 (0.47-0.94)	.02
Cardiology consultation	619 (15.7)	605 (10.5)	0.49 (0.37-0.66)	<.001
Left heart catheterization	113 (2.9)	124 (2.2)	0.48 (0.28-0.83)	.009
Revascularization (PCI or CABG within 30 d)	40 (0.7)	34 (0.9)	0.41 (0.16-1.05)	.06

Abbreviations: AOR, adjusted odds ratio; CABG, coronary artery bypass surgery; ECG, electrocardiography; ED, emergency department; PCI, percutaneous coronary intervention.

### Differences in ED Length of Stay of Discharged Patients

The adjusted median (SE) ED length of stay decreased by 20.2 (5.1) minutes under the accelerated protocol compared with standard care (*P* < .001) (Table 4). Across different quartiles, similar reductions were observed.

**Table 4.** Comparison of Emergency Department LOS Under Accelerated Protocol Compared With Standard Care^a^

Characteristic	LOS by quartile, median (SE), min		
	25%	50%	75%
Accelerated protocol vs standard care	−17.6 (4.4)	−20.2 (5.1)	−8.8 (7.8)
*P* value	<.001	<.001	.27

Abbreviation: LOS, length of stay.

^a^ Covariates used in regression included sex, age, race and ethnicity, time since the initial study start date, history of coronary artery disease, and emergency department site (treated as a random effect).

### Comparison of Resource Utilization Based on Early Rule-Out Under the Accelerated Protocol

We compared patients in the accelerated protocol cohort with those in the standard care cohort based on whether they were ruled out in 0/1 hour or were not ruled out in this time frame (eTable 2 in Supplement 2). Patients who underwent evaluation by the accelerated protocol with MI ruled out within 0/1 hour were more likely to be discharged from the ED (AOR, 1.20; 95% CI, 1.00-1.42), less likely to undergo cardiac stress testing (AOR, 0.55; 95% CI, 0.37-0.82), and less likely to have a cardiology consultation performed (AOR, 0.59; 95% CI, 0.36-0.96) compared with the standard care group. Those in the accelerated protocol cohort who did not have an MI ruled out within 0/1 hour did not have an increase in ED discharges (AOR, 0.90; 95% CI, 0.80-1.01) and had similar reductions in cardiac stress testing (AOR, 0.66; 95% CI, 0.49-0.87) and cardiology consultations (AOR, 0.66; 95% CI, 0.47-0.91).

## Discussion

This secondary analysis of a large, pragmatic randomized clinical trial illustrates the effect of an accelerated ED hs-cTnI protocol on downstream health care resource utilization within a US health care system. Overall, implementing an accelerated protocol was associated with reduced resource utilization. The reductions were seen in cardiac stress testing, cardiology consultations, left heart catheterizations, and ED length of stay. Although data in the US are limited, a study in California recently published similar reductions in cardiac testing using a hs-cTn protocol limited to patients with chest pain.^20^

Implementing the accelerated protocol was not associated with higher adjusted odds of patients being discharged from the ED. This finding differed substantially from European clinical trial data using similar accelerated protocols, which demonstrated adjusted odds of ED discharge as high as 1.6.^9,21^ When we limited our analysis to compare the subset of patients who met early rule-out criteria under the accelerated protocol vs the standard care protocol or those with a chief symptom of chest pain, the adjusted odds of ED discharge were comparable with these European results.

Knowledge of hs-cTnI values below the 99th percentile could theoretically identify more patients for cardiac stress testing, left heart catheterization, and revascularization.^13,14^ Nevertheless, this hypothesis did not bear out in the trial. Any theoretical increase in testing associated with indeterminate hs-cTnI levels was offset by reductions associated with very low levels that qualified for MI being ruled out early. Another study found that the accelerated protocol approach was cost neutral compared with standard care.^22^ There was no statistical difference in overall costs to hospitals or patients.

### Strengths and Limitations

The findings from this study reveal the complexity involved in evaluating these associatons. The study has several strengths. First, the trial focused on patients for whom clinicians made management decisions based on hs-cTnI values below the 99th percentile for the first time in the US. Second, the trial enrolled consecutive patients and focused on a diverse patient population, indicating how hs-cTnI testing is used in the US. The trial did not limit enrollment to patients with a chief symptom of chest pain. Instead, we included patients with any symptoms that prompted evaluation for MI. Hence, most hospital admissions occurred for reasons unrelated to evaluations for MI. Our findings of reduced cardiac resource utilization are modest and should be interpreted in the context of the broad use of hs-cTn testing in the US, regardless of patients’ clinical histories being highly suggestive of MI. An hs-cTn testing approach applied more specifically to patients with clinical symptoms of MI in US EDs could have a more impactful effect on discharge rates and cardiac testing.

Our study also has some limitations. Due to the large cohort size, we relied on a review of local and statewide health information exchanges to capture health care resource utilization. It is possible that this method missed some cardiac testing or procedures. Cardiac stress testing (3.5%) and left heart catheterization (1.1%) procedures occurred infrequently in the overall cohort. However, these numbers were substantially higher when limited to patients with a chief symptom of chest pain. Overlap of our trial with the COVID-19 pandemic may have affected testing availability. These lower rates of cardiac stress testing and left heart catheterization also reflect eligibility restrictions of the trial to patients with hs-cTn values below the 99th percentile. The second half of the stepped-wedge implementation for this trial overlapped with the onset of the COVID-19 pandemic, which posed unforeseen challenges and introduced unexpected confounders. High COVID-19 infection rates during the study period may have also affected our findings by reducing the proportion of patients considered safe for discharge or observation placement. There were also competing COVID-19 demands that limited clinician education on the use of the new 0/1-hour algorithm and may have diluted its implementation and effectiveness.

## Conclusions

In this secondary analysis of a stepped-wedge randomized clinical trial of a 0/1-hour hs-cTnI protocol to rule out MI in the ED, the accelerated protocol was associated with reduced cardiac testing and median ED length of stay. These findings were more pronounced among patients who qualified for early rule-out within 1 hour. Our findings highlight the clinical significance of an accelerated 0/1-hour hs-cTnI protocol in optimizing workflow, improving resource utilization, and enhancing patient flow.

## Notes

Supplement 1.
Trial Protocol and Statistical Analysis Plan

Supplement 2. **eFigure 1.** Stepped-Wedge Clinical Trial Design **eFigure 2.** Myocardial Infarction Exclusion Protocols **eTable 1.** Unadjusted Comparisons of Healthcare Resource Utilization **eTable 2.** Adjusted Odds for Healthcare Resource Utilization Among Patients With or Without 0/1-Hour Rule-Out in the Accelerated Protocol Compared to Standard Care

Supplement 3. **Nonauthor Collaborators.** RACE-IT Research Group members

Supplement 4.
Data Sharing Statement
