# Supplementary material for: Health Care Resource Utilization for Patients With Suspected Myocardial Infarction: A Secondary Analysis of the RACE-IT Randomized Clinical Trial
Source: JAMA Netw Open. 2025 Apr 25;8(4):e256930. doi: 10.1001/jamanetworkopen.2025.6930 (PMC12032557; doi:10.1001/jamanetworkopen.2025.6930)
Supplement: Supplement 1. — Trial Protocol and Statistical Analysis Plan [file jamanetwopen-e256930-s001.pdf]

RACE-IT – Rapid Acute Coronary Syndrome Exclusion using the Beckman Coulter Access high-sensitivity I Troponin.

Principal Investigators: Joseph Miller, MD, MS (Emergency Medicine), James McCord, MD (Cardiology), Bernard Cook, PhD (Pathology)

Co-Investigators: Phillip Levy, MD, MPH (Emergency Medicine, WSU), Shooshan Danagoulian, PhD (Health Economist, WSU), Amy Tang, PhD (Statistician), Simon Mahler, MD (Wake Forest), Nicholas Mills, MD (University of Edinburgh)

STUDY AIM, BACKGROUND, AND DESIGN

Cardiovascular disease is the leading cause of mortality in the United States. Cardiac biomarkers are critical in detecting acute myocardial injury (AMI) amongst those with suspected acute coronary syndrome (ACS). There have been continued improvements in cardiac troponin (cTn) as a biomarker for AMI.[1] Recently, the FDA cleared high-sensitivity cTn (hs-cTn) assays for evaluation of patients with suspected ACS. These highly sensitive and precise assays provide the ability to diagnosis and rule-out AMI earlier in the emergency department (ED).[2-4]

Henry Ford Health System (HFHS) is implementing the Beckman Coulter Access hs-cTnI assay for use throughout its 9 EDs. Within this implementation, HFHS is changing the clinical workflow for the ED evaluation of patients with symptoms suggestive of ACS. This change in clinical workflow, titled the RACE-IT pathway, includes new guidance to stage patients based on hs-cTnI values and send those home that have very low hs-cTnI values. The RACE-IT pathway additionally uses clinical risk stratification with the HEAR score (HEART[5] score without troponin component) to determine which patients with suspected ACS and quantifiable hs-cTnI values below the 99th percentile should be placed in observation for further testing or sent home with a low HEAR score.

This planned implementation of a new clinical pathway using hs-cTnI provides a unique opportunity to measure patient outcomes and clinical processes in a real-world scenario throughout an integrated health system. This measurement will allow for the assessment of the effectiveness of the new pathway in discharging patients safely from the ED. It also allows for assessment of resource utilization in the hospital (e.g. stress testing, coronary CTA, cardiac catheterization, etc). Publication of these results will also provide important data to other health systems considering similar implementation.

Overall Objective: To measure effectiveness and safety of implementing a novel, staged rapid rule-out protocol, which incorporates the Beckman Coulter Access hs-cTnI assay and

the HEAR score, in comparison to an existing 0/3-hour standard of care (SOC) protocol in the Henry Ford Hospital Health System's 9 EDs.

Aim 1. To compare safe ED discharge rates to home among patients receiving SOC evaluation for ACS and the new RACE-IT pathway. We hypothesize that patients evaluated under the new pathway who have ED troponin values <19 ng/L will have higher rates of safe discharge home. A safe discharge constitutes a discharge in which a patient has no MACE over the subsequent 30-days. MACE will be defined as all-cause death or AMI at 30 days.

Aim 2. To create a registry of patients with quantifiable hs-cTnI values that fall below the 99th percentile (3-18 ng/L) that are placed into observation for suspected ACS. This registry will allow for exploratory analysis of phenotypes within this hs-cTnI range that may confer low risk. We will specifically explore patient phenotypes that may be safely discharged based on different hs-cTnI absolute/delta values combined with clinical characteristics such as the HEAR score. We will also evaluate the utilization of stress testing, coronary CTA, and coronary catheterization in this cohort evaluate the added diagnostic value of these tests based on different hs-cTnI cut-offs.

Aim 3. To assess the cost-effectiveness of a 0/1-hour protocol (RACE-IT) compared to SOC evaluation of ACS. We will measure resource utilization, length of stay, rates of admission, hospital payments received, and cardiology consultations within 30-days to compare operational cost and quality associated with patient care under both protocols.

Design Overview. This is a pragmatic, implementation study testing the implications of a real-world execution of a rapid evaluation pathway for suspected ACS using the Beckman hs-cTnI assay. As the new protocol is executed across 9 EDs within an integrated health system, we will study its effects on patient and system-level metrics. A modified stepped wedge design will be utilized that allows comparison of the RACE-IT pathway with SOC management. Each of the 9 centers will have 3 phases: SOC with data collection, implementation phase (RACE-IT pathway started but data is not collected for study purposes), and RACE-IT pathway (RACE-IT active and data is collected for study purposes).The duration of the SOC and RACE-IT phases in the study will vary at each site depending on when they start the new protocol (Figure 1).The implementation phase where data will not be collected for study purposes will last 3 weeks at each site. We estimate that all sites will transition to the new protocol within 7 months and data analysis will be completed within 1 year. The study will evaluate 30-day MACE, inclusive of death or AMI. This study will leverage the Michigan Heath Information Network (MiHIN), which provides robust state-wide follow-up data on all patients. The planned sample size is 11,000 patients.

Primary and Secondary Outcomes. The primary outcome is the proportion of safe discharges home from the ED without death or AMI within 30-days. Secondary endpoints include: 30-day AMI, Death (cardiac and non-cardiac), length of ED stay, hospital payments received for ED and hospital encounters, radiation exposure associated with cardiac testing, rates of hospital admission and observation placement, rates of revascularization procedures (CABG/PCI), rates of cardiology consultation, and survival at 1 year.

Impact. Data on the real-world application of a protocol using a rapid hs-cTnI based evaluation strategy for suspected ACS will have significant implications for patients and health

systems, including patient morbidity, system operations, and cost-effectiveness. Furthermore, assessment of outcomes in patients with minimally elevated hs-cTnI values who require further testing provides rich data to assess if certain patient phenotypes may be safely discharged based on different hs-cTnI delta values or clinical characteristics.

1. SETTING, SUBJECT POPULATION AND ELIGIBILITY

Setting: This study will take place within the HFHS. The system has 9 EDs which together care for approximately 464,000 patient encounters each year. The main hospital ED is a quaternary care, level 1 trauma center in the heart of Detroit and cares for a primarily urban population. Four EDs are parts of community hospitals that lie in suburban settings. These include Henry Ford Allegiance Hospital (Jackson, MI), Henry Ford West Bloomfield Hospital (West Bloomfield, MI), Henry Ford Macomb Hospital (Macomb, MI), and Henry Ford Wyandotte Hospital (Wyandotte, MI). The remaining 4 EDs are free-standing EDs in metro Detroit operating 24/7, including one of the largest free-standing EDs in the nation, our Fairlane ED (Dearborn, MI), which sees nearly 70,000 encounters each year.

Inclusion criteria: Patients ≥18 years old presenting to the ED for whom a treating clinician suspects ACS and orders a baseline ECG and cardiac troponin.

Exclusion criteria:

a) ST-segment Myocardial Infarction (STEMI) leading to immediate reperfusion therapy
b) Any ED-drawn hs-cTnI value > 99th percentile (18 ng/L)
c) Clear traumatic cause for symptoms (e.g., direct chest wall trauma, motor vehicle accident)
d) A transfer from another facility
e) Primary residence outside the state of Michigan
f) Previous inclusion in the study
g) Enrolled in hospice

Rationale: We focus this study on patients for whom the new ACS evaluation protocol is intended for, and not patients with STEMI that require immediate reperfusion. We exclude patients transferred from other hospitals who may have been exposed to other ACS evaluation protocols. We also exclude patients from outside the state of Michigan as 30-day outcome assessment is less reliable in these patients given our reliance on a Michigan health information exchange.

Enrollment and/or Screening

We will identify patients through the electronic health record (EHR), EPIC. Through weekly, standardized data reports during the study period, we will identify all adults across 9 HFHS EDs that had an ECG and serial hs-cTnI testing ordered during their ED evaluation. After applying the above exclusion criteria to these reports, we will use the reports to create a master registry of eligible study patients. For those excluded, we will maintain a log of ineligible patients for standardized reporting. This screening log will include age, sex, and

reason for exclusion.

The volume of eligible ED patients over the course of the planned study period may exceed our necessary sample size. Data collection will include all eligible patients over the study period. We will perform complete data collection on the eligible population for analysis. Further description of sample size planning is described below.

The study population is identified through the master registry that populates as patients are cared for within the health system. The study team will not approach eligible patients during their evaluation in the ED. As the intervention is being implemented as standard practice in the EDs, individual consent will not be sought at ED presentation. The waiving of consent has been done in other similar trials.[6] We have established common data fields in the EHR to capture key data elements described below.

2. STUDY PROCEDURES

Overview

The study will measure the real-world evaluation, treatment, and outcomes of patients managed across 9 EDs with SOC evaluation of ACS compared to the RACE-IT pathway. We first describe the SOC protocol and RACE-IT pathway, and then discuss the implementation strategy and relevant data collection.

SOC Protocol

The existing SOC protocol throughout all 9 HFHS EDs includes evaluation of suspected ACS with 0- and 3-hour ECG and use of the 99th percentile as the upper reference limit for Tn testing. The lab reports actual Tn values above the 99th percentile or simply as < "18 ng/L" if the measurement is below the 99th percentile. Clinicians base discharge decisions from the ED on Tn values below the 99th percentile and a HEAR score <4. Patients with clinical concern for possible ACS and a HEAR score ≥ 4 are typically placed in observation care in the hospital. Patients with very high suspicion of ACS, including unstable angina, may be directly admitted to a cardiology unit. Physicians always have the ability to place patients in observation if the patients have low HEAR scores but residual concern for ACS. Patients with rising hs-cTnI levels above the 99th percentile concerning for AMI are admitted to a cardiology floor. Observation units are managed by medicine hospitalists or by emergency physicians.

RACE-IT Pathway

This pathway includes several stages to guide clinical decision making. Appendix A shows a figure of this pathway. First, if the first hs-cTnI value is <4 ng/L, a patient is eligible for ED discharge. Second, if the initial hs-cTnI value is 4 ng/L and the 1-hour has a lab calculated increase <4 ('delta <4'), the patient is eligible for discharge home. Third, if a patient's initial hs-cTnI is ≥5 and ≤18 ng/L, they are to receive repeat testing at 1 and 3 hours. If all values remain ≤18 ng/L, clinicians are to apply the HEAR score. Those with a HEAR score <4 are eligible for ED discharge, and observation is considered for those with a HEAR score ≥4. The

management of patients with hs-cTnI > 99th percentile (18 ng/L) is no different within the RACE-IT pathway compared to SOC.

Implementation and Data Collection

HFHS will be implementing the RACE-IT pathway across 9 EDs through a staggered process. The ED operations team has chosen to implement in a staggered fashion to allow for adequate education at all sites by clinical leadership. Implementation occurs in 1 ED at a time over the course of 33 weeks until all EDs are using the new pathway. The implementation phase will be 3 weeks in duration. We will collect data for a minimum of 3 weeks prior to a and a minimum of 3 weeks after implementation. No data collection will occur during the implementation phase to allow a large number of ED clinicians to learn the new protocol and change practice accordingly. Clinical operation teams and ED leadership will provide extensive education on the new protocol throughout the IP period. We will track rates of adherence to the RACE-IT pathway for each cluster over the course of the trial. Figure 1 provides an overview of the stepped design.

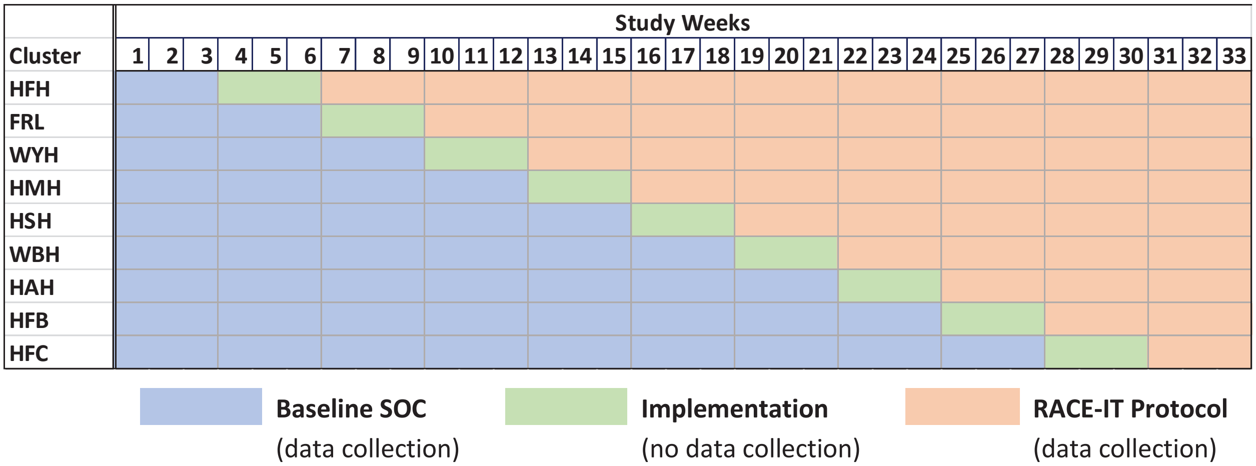

Figure 1. **Stepped Implementation Design Matrix**. Each segment represents a 3-week period. Blue segments indicate enrollment under the standard of care protocol, and orange cells indicate enrollment under RACE-IT pathway. Green cells indicate 3-week implementation periods for clinician education and time to accommodate practice change, during which patient enrollment does not occur.

Data Collection

Data collection will occur in REDCap (Nashville, TN), a secure data management tool that will contain all case report forms (CRFs). Data elements are outlined in Table 1. We will upload data from EPIC reports into REDCap and supplement this data with manual abstraction and data entry. We will obtain most data elements through EPIC reports and through standardized chart review. Prior to study initiation, we will finalize a data dictionary for all variables and train team members on chart abstraction.

Data collection will include standard demographic and clinical data on cardiovascular risk

factors. Measures in the ED will include length of stay, disposition (home, observation, admission), primary and secondary diagnoses, and vital signs. Laboratory data collection will include CBC, metabolic profile, BNP, D-dimer, and hs-cTnI values. Data collection of hs-cTnI values will occur through routine samples sent to each ED's laboratory and tested using Beckman Coulter Access analyzers. While clinicians operating under the SOC protocol will not have access to hs-cTnI values <18 ng/L, the laboratory will report these values to the research team such that we will collect exact hs-cTnI values for all patients in the study. Clinical risk factor data collection will also include the HEAR score. Prior to this study, HFHS has placed prompts in the EHR to standardize documentation of the HEART score. These prompts exist in the medical decision-making section of every ED clinician note for patients with possible symptoms of ACS.

For patients placed in observation, we will enrich data collection with standardize data collection of other cardiac testing with a focus on the presence or absence of ischemic findings on stress testing, EF, coronary artery disease on coronary CT or heart cath, and any revascularization procedure (PCI or CABG). Hospital metrics will additionally include primary and secondary discharge diagnoses, procedural codes for cardiovascular procedures, length of stay, and healthcare charges.

For outcome assessment, we will utilize MiHIN, Michigan's statewide health information exchange (HIE) to assess for MACE events. This HIE provides data directly to HFHS secure data warehouses and is obtained directly from the HFHS analytics team. We will also supplement this data with data provided by the national death index. This HIE provides a comprehensive assessment of any healthcare encounter at an ED or hospital throughout Michigan. The data includes the date and location of the encounter and associated diagnoses. There is the possibility of incomplete reporting or events that occur out of state, though we do not anticipate a bias in outcome assessment between patients managed with SOC and the RACE-IT pathway due to his limitation.

Our team will use the HIE as the primary source for initial evaluation of 30-day MACE for patients that were discharged from the ED or placed in observation during their index encounter. If a patient has no encounters within the HIE over 30-days after their index ED encounter, we will consider that patient to have no event during that time period. For patients who have an encounter identified on the HIE that has no cardiovascular related diagnoses, we will also consider that patient to have no MACE. For patients that have a cardiovascular related diagnosis, our team will supplement the HIE information with any additional EHR information as available. On the 30-day CRF, the team will document the diagnoses associated with the encounter and upload a PDF of the ED note, hospital discharge summary, and cardiac testing for evaluation by the adjudication team. We will redact any patient identifiers prior to this upload. The adjudication team (described below) will use this data to determine if MACE occurred within 30-days. Finally, we will supplement the above processes with the national death index to determine if any death occurred in the study population outside available records.

While MACE are the primary outcome considerations, we will also record all available revascularization procedures (PCI or CABG) and any rehospitalization related to cardiovascular disease over 30-days. Cardiovascular rehospitalization includes readmission

for coronary revascularization, peripheral artery disease, cerebrovascular accidents, congestive cardiac failure without AMI, and atrial and ventricular arrhythmias. Lastly, we will continue to track these outcomes through the HIE out to 1 year following each patient's initial encounter.

Table 1. Core Data Elements

**Demographics**	Age, Sex, Race, Ethnicity
**ED Measures**	Triage time, blood draw time, length of stay, disposition, BP, HR, BMI, diagnoses
**Risk Factors**	HTN, DM, dyslipidemia, tobacco use, CAD, prior MI, PVOD, CKD, CHF, revascularization
**Lab and Imaging**	All hs-cTnI values, BNP, CBC, metabolic profile, d-dimer, CT chest
**Historical Factors**	HEAR score, recorded time of symptom onset, ASCVD score
**Observation Stay Evaluation**	Echo, Stress test, heart cath, CT coronary results, cardiac MRI, cardiology consultation, diagnoses, length of stay
**Hospital Factors**	Length of stay, Revascularization (PCI or CABG), diagnoses
**Outcomes within 30-Days**	Death (cardiac or non-cardiac), AMI, hospital payments received (patient level reimbursement), rehospitalization for cardiovascular disease, non-cardiac diagnoses
**Outcomes, 1-year**	Death (cardiac or non-cardiac), AMI, hospital payments received (patient level reimbursement), rehospitalization for cardiovascular disease

Data relevant to cost-effectiveness analyses includes patient-level reimbursement data from HFHS for each ED visit, observation stay, or hospital admission. This data also includes resource utilization specific to cardiovascular evaluation: echo, stress tests, heart cath, CT coronary results, cardiac MRI, revascularization procedures (PCI or CABG), and cardiology consultation. We will collect CPT codes for all such procedures regardless of whether they were performed in the ED, observation unit or inpatient unit. We will also collect data on length of stay in the ED, observation unit, and hospital.

Main Outcome Adjudication

We have convened an independent adjudication committee that will determine the primary outcome of MACE. Two independent adjudicators will review each case, and if there is disagreement, a third adjudicator will review as well. All adjudicating physicians will be board-certified cardiologists or emergency medicine physicians. For the diagnosis of AMI, they will determine it in accordance with the fourth Universal Definition after review of all available 30-day clinical data and the serial individual hs-cTnI measurements utilized during the index or subsequent visit. Each adjudicator will independently use their expert opinion to assess if the requirements of an AMI adjudicated diagnosis were met, including the presence of a clinically significant rise and/or a fall of hs-cTnI levels. In the event that adjudicating physicians feel that too much data is missing to make an adequate determination for suspected MACE, we will

code the outcome as unknown and incorporate into a sensitivity analysis as outlined below.

Protocol Implementation Metrics

Throughout the stepped-wedge protocol, we will track ED operational metrics to assess uptick of clinician use of the RACE-IT pathway. These metrics include weekly rates by site of: hs-cTnI ordering that is consistent with the pathway, discharge and admission/observation rates, and documentation of the HEAR score.

3. Data Analysis and Statistical Considerations

Primary Analysis

The primary outcome analysis compares rates of safe ED discharge among patients receiving SOC vs. RACE-IT pathway. We hypothesize that there will be a higher rate safe ED discharge under the RACE-IT pathway compared to SOC. Generalized mixed models will be used to evaluate the effect of RACE-IT to account for the clustering of patients within centers, where center will be included as a random effect.[7] The primary analysis also will adjust for the baseline variables listed in Table 1 (age, sex, race, and comorbidities), which are known affect cardiovascular outcomes. We will include a time-dependent variable to denote the change from SOC to the RACE-IT protocol phase as determined by the stepped wedge design and include time as an independent variable in all models. The effect of implementation of the RACE-IT protocol will be presented as an odds ratio and its 95% confidence interval (CI).

The statistical nature of missing data will be studied, and sensitivity analyses will be proposed to analyze the impact of missing data on results and propose the most appropriate imputation method. Sensitivity analyses include an analysis to assume the worse that patients deemed to have an unknown primary outcome by the adjudication committee have MACE within 30-days. The primary analysis will be performed according to the intention-to-treat principle. All analyses will use SAS version 9.4 (SAS Institute Inc., Cary, NC). All tests will be two-sided with p-value < 0.05 as statistical significance.

Secondary Analyses

We will also compare 30-day MACE rates (binary outcome) among patients discharged home or placed in observation under the SOC vs. RACE-IT protocols. For this analysis, we will again use generalized linear mixed models to evaluate the effect of RACE-IT to account for the clustering of patients within centers, where center will be included as a random effect.

Using outcome assessment to 1-year, we will also compare survival curves between patients in the SOC or RACE-IT cohorts. If patients have no AMI or death, they will be censored 1-year following their index encounter. If they have AMI or death, they will be censored at the time of that initial event. Survival curves will additionally look at rates of revascularization (CABG or PCI) and rehospitalization for cardiovascular disease. The time-to-event curves will be

calculated with the Kaplan-Meier method and compared, when appropriate, using marginal Cox proportional hazards regression model. Multilevel and multivariable Cox regression models are considered using the same patient covariates as the primary analysis and incorporating exposure to SOC or the RACE-IT pathway.

For Aim 2, we perform exploratory analyses on patients placed in observation both under SOC and RACE-IT. We will compare utilization of stress tests, cardiology consultation, coronary CTA, coronary angiography, and PCI between SOC and RACE-IT. We also will perform sensitivity analyses to determine if phenotypes based on HEAR score, age, sex, race, and different hs-cTnI value/delta cut-offs are associated with low rates of MACE at 30-days. We will use a similar mixed model for these analyses as described for the primary outcome above.

Sample Size Considerations

Power and sample size are calculated based on a cluster randomized stepped wedge design (PASS 2019). Intraclass correlation coefficient (ICC) is set to 0.05, and we will have 9 steps with 1 cluster each. We assume the following additional variables: SOC patients will have a safe discharge rate of 40% and RACE-IT patients will have a rate of 45%. Assuming variable cluster size (coefficient of variance 0.4), an alpha of 0.05, and 90% power, we estimate that a sample size of 11,070 patients will need to be evaluated to test our primary endpoint. While allowing for variable cluster sizes, this sample size equates to an average of 123 patients per cluster per time period (90 total 3-week block time periods). We will include all patients in each time period over the course of the trial, and we anticipate exceeding the requisite sample size.

Cost-Effectiveness.

To assess the cost-effectiveness of a RACE-IT pathway compared to the SOC protocol, we will measure resource utilization, length of stay, rates of admission, MACE, and hospital charges at 30-days to compare operational cost and quality associated with patient care under both protocols. Resource utilization will include detailed CPT code information on all performed cardiovascular procedures both in the ED and hospital setting (regardless of whether a patient is discharged home from the ED or brought into observation or inpatient care.

Within-trial incremental costs associated with the SOC and RACE-IT protocols will be estimated using hospital reported reimbursements from insurers received for ED and inpatient hospital costs. Incremental cost effectiveness will be defined with respect to the primary clinical outcome, either as avoided adverse outcomes or improved patient outcomes. The length of study does not allow a trial-based evaluation of the quality-adjusted life years (QALYs). However, we will use the incidence of AMI and other adverse outcomes combined with estimates from literature to compute the expected QALY for rate of occurrence of such outcomes. We plan nonparametric bootstrapping estimates of mean difference in cost and effects (events avoided and QALY gained) from patient-level data to account for uncertainty due to sampling variation in cost-effectiveness. We will incorporate relevant data sources and literature to extrapolate the longer-term costs and benefits derived from observed differences

in cardiovascular events to generate full estimates of the cost-effectiveness of the new protocol.

Additional Analyses

Additional analyses will compare ED length of stay, hospital length of stay, and imaging/procedure utilization between patients in the SOC vs. RACE-IT protocols. Such procedures include stress tests, echo, CTA, coronary angiography, and cardiac MRI (as described above). Also included is total radiation exposure associated with cardiac testing.

Trial Pause Contingency

Due to the possibility of research disruptions due to COVID-19, we have planned contingency rules for pausing implementation and collection of data on eligible patients. A pause will occur if research administration for the health system places a hold on clinical research or if ED weekly patient encounters throughout all EDs fall >25% from pre-trial levels. Trial activities will resume once any associated research administration restrictions are lifted and weekly encounters are within 25% of pre-trial rates.

4. BIOBANKING

Throughout the trial, we will biobank 1-2 aliquots of serum on patients that meet the above trial eligibility criteria. Dr. Cook will oversee these procedures. Biobanking will occur through an automated process in the lab that sets aside tubes that were used for hs-cTnI analysis. Lab personnel will aliquot remaining serum from these tubes (approximately 0.5 – 2 mL) for storage. Patients will not be exposed to an added blood draw for the purpose of this biobanking – only serum that would otherwise be wasted will be used. Once aliquoted, we will store serum at -80^{0}C for future exploratory analyses.

5. ANTICIPATED RISKS

We are measuring the effects of a change in routine medical care. The change falls within an overall quality improvement project and does not constitute a research intervention. Hence, the anticipated risk for patients participating in data collection during this transition in usual clinical care is a loss of confidentiality.

6. ANTICIPATED BENEFITS

Patients that are seen in the ED for suspected ACS may benefit from changes in routine clinical care brought about through implementation of the new clinical pathway. These benefits could include higher rates of safe ED discharge and reduced need for invasive cardiac test.

7. RENUMERATION/COMPENSATION

No remuneration will occur in this study.

8. COSTS

Patients have no costs related to study involvement.

9. CONSENT PROCESS AND DOCUMENTATION

For assessment of this protocol implementation, we request waiver of written consent. The purpose of this analysis is to assess the safety and efficacy of implementing a new operational protocol within the health system. All patients that present with symptoms concern for ACS to the included EDs will undergo usual care evaluation either under the prior SOC or new ACS pathway as our ED operations team roll-out the protocol in a staggered manner.

Data collection occurs through patient information available in the EHR that is populated during usual clinical care. This includes data available in EPIC and through the HFHS access to Michigan HIE data.

10. PRIVACY AND CONFIDENTIALITY

To protect privacy and confidentiality, all data will be stored in secure and encrypted servers within HFHS. We will assign each patient an anonymous study ID within our secure REDCap server. We will store a study spreadsheet that links patient name and MRN to this study ID within a secure HFHS drive. All drives and servers are encrypted and behind the HFHS firewall. We will maintain this spreadsheet for 2 years following study completion in the event that any audit is necessary and then destroy this data link. All research team members that have access to patient records will have appropriate training in good clinical practice and best practices for data security. Access will be limited to only those listed on this IRB.

11. DATA AND SAFETY MONITORING PLAN

A lead research coordinator will perform regular data quality checks in REDCap after every 1000 patients have data entered. This coordinator will report to the study executive team to remedy any problems that arise with data quality. The study executive team will report any data breaches involving PHI directly to the IRB. While a formal data and safety monitoring committee is not appointed because this is not an interventional trial, any determination of harm through system implementation of the new operational protocol will be communicated directly to the operations leadership and chairs of Emergency Medicine and Cardiology. This operational leadership will determine changes or termination of the new protocol.

12. QUALIFICATIONS OF THE INVESTIGATOR(S)

Drs. Miller, McCord, and Cook form the study executive team and have extensive experience leading clinical trials. Dr. McCord has been an investigator in numerous studies involving ED based cardiac care. He has multiple publications in this area. Dr. Miller is routinely involved in ED based clinical trials, particularly in neurovascular and cardiovascular care. Dr. Cook has a wealth of laboratory research experience and has worked closely with Beckman Coulter in incorporating their assay into the HFHS.

13. APPENDIX A. RACE-IT Pathway

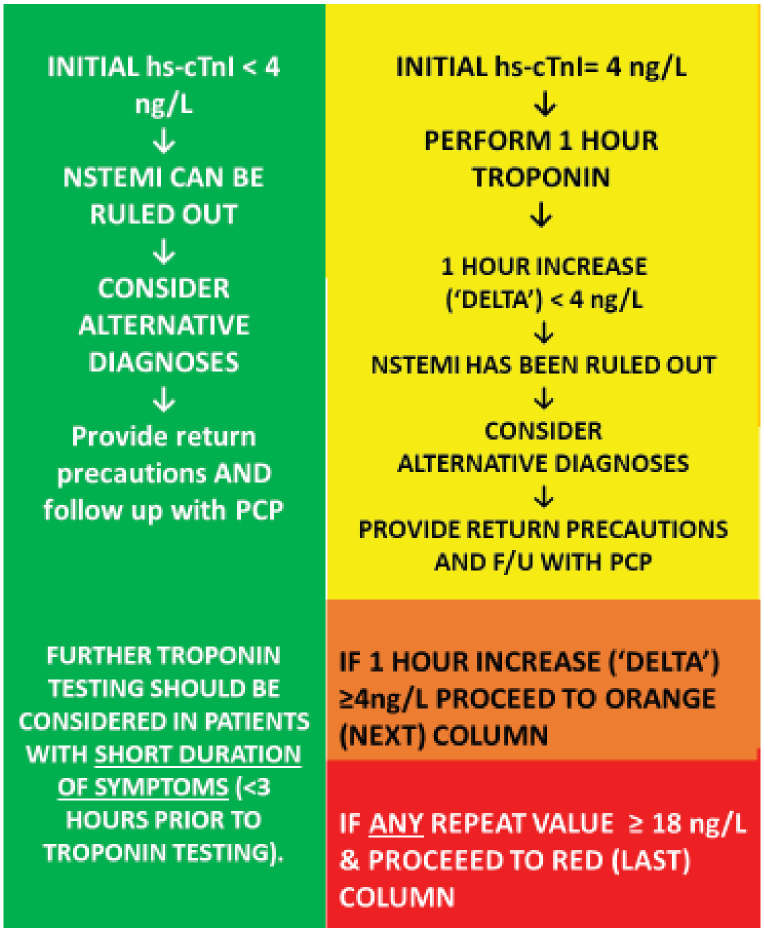

INITIAL hs-cTnI ≥5 but <18ng/L

↓

REPEAT troponins at 1 HOUR AND 3 HOUR

↓

ALL VALUES REMAIN < 18 ng/L

↓

NSTEMI HAS BEEN RULED OUT

HEAR ≤3	HEAR ≥4
Provide return precautions and F/U with PCP	Consider observation placement for further evaluations

'HEAR SCORE'
IS IDENTICAL TO 'HEART' SCORE BUT CAN ONLY BE APPLIED TO PATIENTS WITH ALL TROPONIN <99TH PERCENTILE (18 ng/dL)

IF ANY REPEAT VALUE ≥ 18 ng/L PROCEEED TO RED (LAST) COLUMN

ANY hs-cTn I ≥18 ng/L
(99th percentile)

NSTEMI type 1 or type 2 diagnosis requires at least 1 hs-cTnI value above 99th percentile (18 ng/L) with a typical rise and/or fall in the hs-cTnI measurements and should be supported by at least 1 of the following (if none of these are present the diagnosis is acute cardiac injury):
- clinical ischemic symptoms
- ECG findings
- cardiac imaging results

Although NSTEMI may be diagnosed with any hs-cTnI value ≥ 18ng/L, levels ≥ 100 ng/L have been designated as the HFHS 'CRITICAL VALUE', which triggers lab notification to the treating physician

An Increase/decrease in hs-cTnI from initial value by ≥ 15 ng/L ('delta' ≥ 15ng/L) at 1 hour increases the likelihood of a NSTEMI (either acute if rising or subacute if falling)

Minimal increase/decrease from intial value by <15 ng/L (aka 'delta <15') at 1 hour is more likely the result of chronic cardiac injury.

REFERENCES

1. Bularga A, Lee KK, Stewart S, et al. High-Sensitivity Troponin and the Application of Risk Stratification Thresholds in Patients With Suspected Acute Coronary Syndrome. Circulation 2019;140:1557-68.
2. Chapman AR, Hesse K, Andrews J, et al. High-Sensitivity Cardiac Troponin I and Clinical Risk Scores in Patients With Suspected Acute Coronary Syndrome. Circulation 2018;138:1654-65.
3. Christenson RH, Duh SH, Apple FA, et al. Pivotal Findings for a High-Sensitivity Cardiac Troponin Assay: Results of the HIGH-US Study. Clin Biochem 2019.
4. Hollander JE. High-Sensitivity Troponin: Time to Implement. Ann Emerg Med 2018;72:665-7.
5. Laureano-Phillips J, Robinson RD, Aryal S, et al. HEART Score Risk Stratification of Low-Risk Chest Pain Patients in the Emergency Department: A Systematic Review and Meta-Analysis. Ann Emerg Med 2019;74:187-203.

Version Date: 5 JULY 2020
Version #: 1.0

6. Lee C, Greene SL, Wong A. The utility of droperidol in the treatment of cannabinoid hyperemesis syndrome. Clin Toxicol (Phila) 2019;57:773-7.
7. Hussey MA, Hughes JP. Design and analysis of stepped wedge cluster randomized trials. Contemp Clin Trials 2007;28:182-91.
