# Supplementary material for: Health Care Resource Utilization for Patients With Suspected Myocardial Infarction: A Secondary Analysis of the RACE-IT Randomized Clinical Trial
Source: JAMA Netw Open. 2025 Apr 25;8(4):e256930. doi: 10.1001/jamanetworkopen.2025.6930 (PMC12032557; doi:10.1001/jamanetworkopen.2025.6930)
Supplement: Supplement 2. — eFigure 1. Stepped-Wedge Clinical Trial Design eFigure 2. Myocardial Infarction Exclusion Protocols eTable 1. Unadjusted Comparisons of Healthcare Resource Utilization eTable 2. Adjusted Odds for Healthcare Resource Utilization Among Patients With or Without 0/1-Hour Rule-Out in the Accelerated Protocol Compared to Standard Care [file jamanetwopen-e256930-s002.pdf]

Supplementary Online Content

Miller J, Cook B, Gunaga S, et al. Health care resource utilization for patients with suspected myocardial infarction: a secondary analysis of the RACE-IT randomized clinical trial. *JAMA Netw Open.* 2025;8(4):e256930.
doi:10.1001/jamanetworkopen.2025.6930

eFigure 1. Stepped-Wedge Clinical Trial Design
eFigure 2. Myocardial Infarction Exclusion Protocols
eTable 1. Unadjusted Comparisons of Healthcare Resource Utilization
eTable 2. Adjusted Odds for Healthcare Resource Utilization Among Patients With or Without 0/1-Hour Rule-Out in the Accelerated Protocol Compared to Standard Care

This supplementary material has been provided by the authors to give readers additional information about their work.

© 2025 Miller J et al. *JAMA Network Open.*

eFigure 1. Stepped-Wedge Clinical Trial Design

Abbreviations: UHB=Urban Hospital-based, SHB= Suburban Hospital-based, SFS=Suburban Free-standing ED

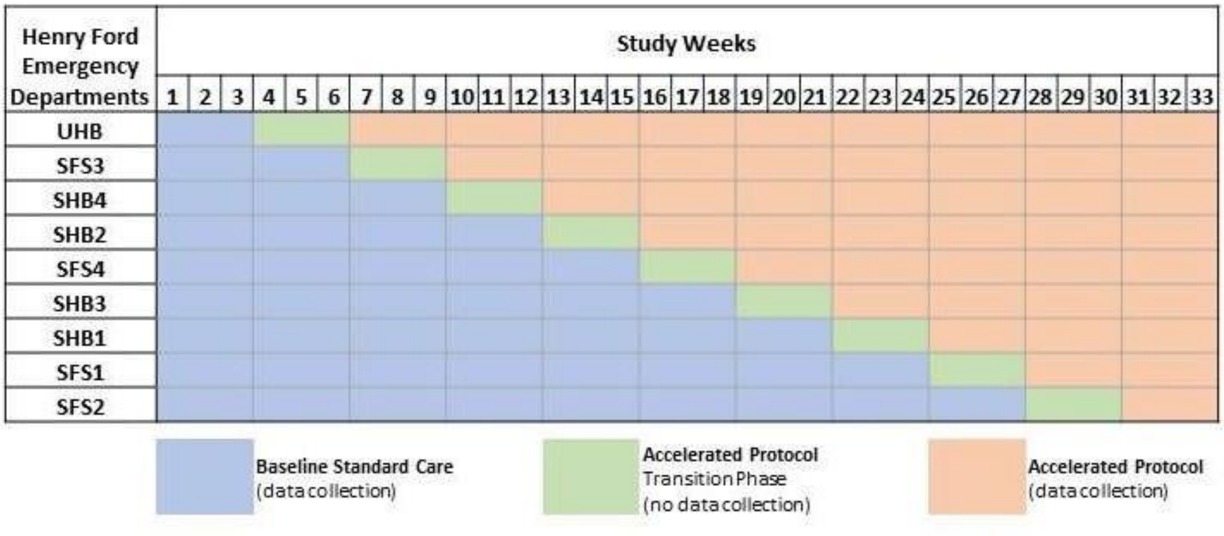

© 2025 Miller J et al. *JAMA Network Open.*

eFigure 2. Myocardial Infarction Exclusion Protocols

Abbreviations: hs-cTnI=high-sensitivity cardiac troponin I, hr=hour.

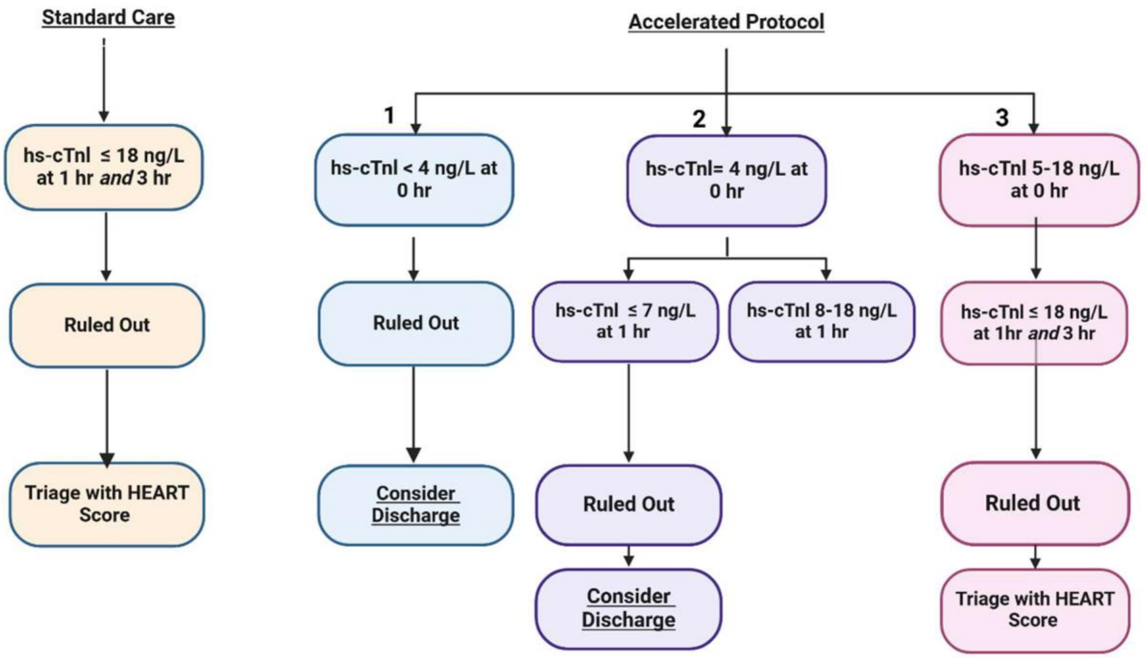

*****The protocol advised that further hs-cTnI testing should be considered in those that present < 3 hours after symptom onset but were not mandated.

© 2025 Miller J et al. *JAMA Network Open.*

eTable 1. Unadjusted Comparisons of Healthcare Resource Utilization

**Outcomes,** No. (%)	**Overall** N = 32,608	**Standard Care** N = 13,505	**Accelerated Protocol** N = 19,103	**Unadjusted Odds Ratio (95% CI)**	**p- value**
**ED Disposition**					
Discharged	19152 (58.73%)	8070 (59.76%)	11082 (58.01%)	1.01 (1.04, 1.16)	p<0.001
Inpatient	9148 (28.05%)	3529 (26.13%)	5619 (29.41%)		
Observation	4308 (13.21%)	1906 (14.11%)	2402 (12.57%)		
**Cardiac Stress Testing**	1149 (3.52%)	526 (3.89%)	623 (3.26%)	0.64 (0.56, 0.72)	p<0.001
Exercise ECG	30 (0.09%)	17 (0.13%)	13 (0.07%)	0.55 (0.26, 1.16)	p=0.115
Stress Echocardiography	288 (0.88%)	149 (1.10%)	139 (0.73%)	0.51 (0.40, 0.66)	p=0.005
Nuclear Imaging	846 (2.59%)	369 (2.73%)	477 (2.50%)	0.68 (0.59, 0.79)	p<0.001
**Cardiology Consultation**	3291 (10.09%)	1651 (12.23%)	1640 (8.59%)	0.61 (0.56, 0.66)	p<0.001
**Coronary Computed Tomography**	15 (0.05%)	9 (0.07%)	6 (0.03%)	0.47 (0.47.0.47)	p<0.001
**Left Heart Catheterization**	365 (1.12%)	167 (1.24%)	198 (1.04%)	0.80 (0.64, 1.00)	p=0.047
**Revascularization**[B]	113 (0.35%)	50 (0.37%)	63 (0.33%)	0.87 (0.59, 1.28)	p=0.258

A. aOR, adjusted odds ratio; 95% CI, 95% confidence interval.
B. Inclusive of percutaneous coronary intervention or coronary artery bypass surgery

© 2025 Miller J et al. *JAMA Network Open.*

eTable 2. Adjusted Odds for Healthcare Resource Utilization Among Patients With or Without 0/1-Hour Rule-Out in the Accelerated Protocol Compared to Standard Care

Outcome	**0/1-Hour Rule Out** aOR (95% CI)	p-value	**Not Ruled Out at 0/1-Hours** aOR (95% CI)	p-value
**Discharged from ED**	1.2 [1.0, 1.42]	p = 0.046	0.90 [0.80, 1.01]	p = 0.07
**Cardiac Stress Testing**	0.55 [0.37, 0.82]	p = 0.003	0.66 [0.49, 0.87]	p = 0.004
**Cardiology Consultation**	0.59 [0.36, 0.96]	p = 0.03	0.66 [0.47, 0.91]	p = 0.01
**Left Heart Catheterization**	0.71 [0.09, 5.35]	p = 0.74	0.71 [0.32, 1.56]	p = 0.39
**Revascularization (PCI or CABG within 30 days)**	0.74 [0.28, 1.92]	p = 0.53	0.69 [0.43, 1.1]	p = 0.12

*PCI, percutaneous coronary intervention; CABG, coronary artery bypass surgery

© 2025 Miller J et al. *JAMA Network Open.*
