# Supplementary material for: Health Care Resource Utilization for Patients With Suspected Myocardial Infarction: A Secondary Analysis of the RACE-IT Randomized Clinical Trial
Source: JAMA Netw Open. 2025 Apr 25;8(4):e256930. doi: 10.1001/jamanetworkopen.2025.6930 (PMC12032557; doi:10.1001/jamanetworkopen.2025.6930)
Supplement: Supplement 3. — Nonauthor Collaborators. RACE-IT Research Group members [file jamanetwopen-e256930-s003.pdf]

Supplemental Online Content: Nonauthor Collaborators
*First name, last name, and suffix (if applicable) are required and will appear in PubMed.

*Group Name(s): RACE-IT Research Group							
*First Name and Middle Initial(s)	*Last Name	*Suffix (eg, Jr, III)	Academic Degrees	Institution	Location (city, state/province, country)	Role or Contribution, eg, chair, principal investigator	Group (if more than 1 Group listed in the byline) and/or Subgroup (eg, Steering Committee)
Ahmed	Oudeif		MD	Corewell Health			RACE-IT Research Group
Shane	Bole		BS	Henry Ford Health			RACE-IT Research Group
Jacob	Tuttle		MS	Henry Ford Health			RACE-IT Research Group
Aaron	Lewandowski		MD	Henry Ford Health			RACE-IT Research Group
Ayman	Alsaadi		MD	Henry Ford Health			RACE-IT Research Group
Elian	Abou Asala		MD	Cleveland Clinic			RACE-IT Research Group
Nicole	Xu		MD	Advocate Christ Medical Center			RACE-IT Research Group
Sophie	Wittenberg		MD	Henry Ford Health			RACE-IT Research Group
Shazil	Mahmood		MD	Corewell Health			RACE-IT Research Group
Andrew	Schock		MD	The University of Texas Health Science Center at Houston			RACE-IT Research Group
Nicholas	Konowitz		MD	Henry Ford Health			RACE-IT Research Group
Joshua	Fuchs		DO	Henry Ford Health			RACE-IT Research Group
Kate	Joyce		MD	Henry Ford Health			RACE-IT Research Group
Mustafa	Mohammed		MD	Henry Ford Health			RACE-IT Research Group
Ahmed	Kazem		MD	Baylor Scott & White Heart Hospital			RACE-IT Research Group
Kelly	Malette		MD	Corewell Health			RACE-IT Research Group
Gulmohar	Singh-Kucukarslan		MD	Baylor University			RACE-IT Research Group
Jacob	Babel		DO	Henry Ford Health			RACE-IT Research Group
Andrew	Broome		MD	Henry Ford Health			RACE-IT Research Group
Elizabeth	Shaheen		MD	Henry Ford Health			RACE-IT Research Group
Gale	Darnell		MD	Henry Ford Health			RACE-IT Research Group
Gregory	Muller		DO	Santa Clara Valley Medical Center			RACE-IT Research Group
Gust	Bills		DO	Henry Ford Health			RACE-IT Research Group
Jason	Vieder		DO	Corewell Health			RACE-IT Research Group
Steven	Rockoff		MD	Henry Ford Health			RACE-IT Research Group
Brian	Kim		MD	Henry Ford Health			RACE-IT Research Group
Anthony	Colucci		DO	Henry Ford Health			RACE-IT Research Group

Supplemental Online Content: Nonauthor Collaborators
*First name, last name, and suffix (if applicable) are required and will appear in PubMed.

***First Name and Middle Initial(s)**	***Last Name**	***Suffix (eg, Jr, III)**	Academic Degrees	Institution	Location (city, state/province, country)	Role or Contribution, eg, chair, principal investigator	Group (if more than 1 Group listed in the byline) and/or Subgroup (eg, Steering Committee)
Elizabeth	Plemmons		MD	Henry Ford Health			RACE-IT Research Group
Kutiba	Tabbaa		MD	HCA Florida Healthcare			RACE-IT Research Group
