# Supplementary material for: Health Care Resource Utilization for Patients With Suspected Myocardial Infarction: A Secondary Analysis of the RACE-IT Randomized Clinical Trial
Source: JAMA Netw Open. 2025 Apr 25;8(4):e256930. doi: 10.1001/jamanetworkopen.2025.6930 (PMC12032557; doi:10.1001/jamanetworkopen.2025.6930)
Supplement: Supplement 4. — Data Sharing Statement [file jamanetwopen-e256930-s004.pdf]

Data Sharing Statement

Miller. Health Care Resource Utilization for Patients With Suspected Myocardial Infarction.
JAMA Netw Open. Published April 25, 2025. doi:10.1001/jamanetworkopen.2025.6930

Data
Additional Information: RACE-IT ClinicalTrials.gov number, NCT04488913.
Data available: No
